# Aspheric versus Spherical Posterior Chamber Intraocular Lenses

**Published:** 2010-10

**Authors:** Mohammad-Reza Jafarinasab, Sepehr Feizi, Ahmad-Reza Baghi, Hossein Ziaie, Mehdi Yaseri

**Affiliations:** Ophthalmic Research Center, Shahid Beheshti University of Medical Sciences, Tehran, Iran

**Keywords:** Intraocular Lens, Sensar, Akreos AO, Tecnis, AcrySof IQ, Spherical Aberration, Contrast Sensitivity Function, Phacoemulsification

## Abstract

**Purpose:**

To compare spherical aberration and contrast sensitivity function following implantation of four different foldable posterior chamber intraocular lenses (IOLs), namely Sensar, Akreos AO, Tecnis, and AcrySof IQ.

**Methods:**

In this randomized clinical trial, 68 eyes of 68 patients with senile cataracts underwent phacoemulsification and IOL implantation with Sensar (n=17), Akreos AO (n=17), Tecnis (n=17), or AcrySof IQ (n=17). Uncorrected visual acuity (UCVA) and best spectacle-corrected visual acuity (BSCVA), spherical aberration and contrast sensitivity function (CSF) were compared among the study groups, 3 months after surgery.

**Results:**

There was no significant difference between the study groups in terms of age (P = 0.21). Mean postoperative BSCVA with Sensar, Akreos AO, Tecnis, and AcrySof IQ was 0.15±0.10, 0.12±0.9, 0.08±0.08, and 0.08±0.07 logMAR, respectively (P=0.08). Spherical aberration measured over a 4 mm pupil was significantly higher with Sensar and Akreos AO than the two other IOLs. The difference between Tecnis and AcrySof IQ was significantly in favor of the former IOL. Over a 6 mm pupil, spherical aberrations were comparable with Sensar and Akreos AO, furthermore spherical aberration was also comparable among eyes implanted with Akreos AO, AcrySof IQ, and Tecnis. Sensar yielded significantly inferior results as compared to Acrysof IQ and Tecnis. CSF with Sensar was inferior to the three aspheric IOLs at the majority of spatial frequencies. Tecnis yielded significantly better mesopic CSF at 1.5 and 3 cycles per degree spatial frequencies.

**Conclusion:**

Tecnis and AcrySof IQ provided significantly better visual function as compared to Sensar and Akreos AO, especially with smaller pupil size. However, this difference diminished with increasing pupil size.

## INTRODUCTION

Advances in surgical techniques, as well as the invention of new intraocular lens (IOL) materials and designs, have increased expectations from cataract surgery to beyond simply providing good visual acuity. Enhancing visual function and protecting the retina against light toxicity after cataract surgery have become major concerns.^1^

The cornea induces some degree of positive spherical aberration which is compensated by the negative spherical aberration of the clear crystalline lens. However, this compensation gradually decreases as the crystalline lens ages and particularly after cataract extraction and intraocular lens implantation.^2^ Conventional spherical IOLs add positive spherical aberration to the pre-existing aberrations caused by the cornea, increasing the total spherical aberration of the eye.^1^–^3^ One key factor contributing to postoperative spherical aberration is IOL design which has undergone dramatic changes to compensate for the positive corneal spherical aberration.^4^,^5^

The aim of the present study is to determine to what extent spherical aberration induced by a spherical IOL (Sensar AR40e, Advanced Medical Optics Inc., Santa Ana, CA, USA) can be avoided using aspheric IOLs, namely the Akreos AO MI-60 (Bausch & Lomb Inc., Rochester, NY, USA), Tecnis ZA9003 (Advanced Medical Optics Inc., Santa Ana, CA, USA), and AcrySof IQ SN60WF (Alcon Laboratories Inc., Fort Worth, TX, USA). Contrast sensitivity function was also measured and compared among the study groups.

## METHODS

In this double-blind randomized clinical trial, patients aged 50 to 75 years with senile cataracts scheduled for surgery were randomly implanted with the Sensar (n=17), Akreos AO (n=17), Tecnis (n=17), or AcrySof IQ (n=17) IOLs. The number of participants was chosen on the basis of α (error) = 5%, 1-β (power) = 95%, S (maximum standard deviation) = 1.96, and d (least difference in means) = 0.5. Randomization was achieved using a random balanced block. Inclusion criteria were expected postoperative best spectacle-corrected visual acuity of 20/30 or better, preoperative corneal astigmatism less than 1.5 D, axial length between 22.5 and 24.9 mm, and absence of concomitant ocular pathologies. Exclusion criteria were any ocular diseases including corneal opacities or irregularity, dry eye, amblyopia, glaucoma, and retinal abnormalities. Furthermore, cases were excluded from the study in case of IOL tilt or decentration, surgical complications or loss to follow-up. Institutional Review Board approval was obtained and informed consent was signed by all participants after explaining the nature of the study.

IOL calculation was performed using IOL Master (Carl Zeiss Meditec Inc., Dublin, CA, USA) and the target refraction was emmetropia. All patients were operated by a single anterior segment surgeon (MRJ) under retrobulbar anesthesia. A self-sealing 2.8 mm temporal clear cornea incision was created. In case of corneal astigmatism, the incision was made on the steep meridian. A central circular capsulorrhexis measuring 5.0 to 5.5 mm in diameter was performed intending circular overlap of the IOL optic by the capsular rim. Phacoemulsification was performed with the Sovereign phacoemulsification machine (WhiteStar, version 6.0 software, Advanced Medical Optics Inc., Santa Ana, CA, USA), using the divide and conquer technique. All IOLs were implanted within the capsular bag and the incisions were secured without sutures. Postoperatively, the participants were treated with 0.5% chloramphenicol eye drops four times a day for 10 days, and 0.1% betamethasone eye drops six times a day gradually tapered over 6 weeks. All subjects were examined 1, 3, 7 and 21 days, and 2 and 3 months after surgery. Follow-up examinations consisted of measurement of uncorrected visual acuity (UCVA) and best spectacle-corrected visual acuity (BSCVA), slitlamp examination and intraocular pressure measurement. At the final examination (month 3), contrast sensitivity function under photopic and mesopic conditions, and spherical aberration over 4 mm and 6 mm pupils were evaluated.

### Contrast Sensitivity Measurement

Monocular contrast sensitivity function (CSF) was measured with sine-wave gratings at 1.5, 3, 6, 12, and 18 cycles per degree (cpd) spatial frequencies under mesopic (5 cd/m^2^) and photopic (85 cd/m^2^) conditions using the Metrovision Moniteur Ophtalmologique “STATphot” program (Metrovision, Pérenchies, France). During CSF measurement, the chart was viewed from a distance of 2.0 m with the patient’s full correction in place. The log base 10 values were used to statistically analyze each tested frequency.

### Wavefront Evaluation

After instillation of cyclopentolate 1% eye drops and when pupil diameter exceeded 6 mm, aberrometry was performed using the Zywave II aberrometer with Zywave software version 5.2 (Bausch & Lomb, Rochester, NY, USA) in a dark room. This aberrometer was used to calculate spherical aberration over 4 mm and 6 mm pupils in terms of Zernike coefficients.

### Data Analysis

Variables including age, UCVA, BSCVA, CSF, and spherical aberration were expressed in mean ± standard deviation. Postoperatively, quantitative variables were compared between the study groups using the ANOVA test and within each group using paired t-test. SPSS version 13.0 (SPSS Inc., Chicago, IL, USA) was used for analysis with significance set at 5%.

## RESULTS

Sixty-eight eyes of 68 patients (39 male) were randomly implanted with Sensar (n=17), Akreos AO (n=17), Tecnis (n=17), and AcrySof IQ (n=17) IOLs. Mean patient age was 61.1±8.6 (range, 51 to 75) years in the Sensar group, 60.2±3.5 (range, 52 to 73) years in the Akreos AO group, 58.2±6.1 (range, 50 to 70) years in the Tecnis group, and 57.2±5.7 (range, 50 to 71) years in the AcrySof IQ group (P=0.21). Mean postoperative UCVA was 0.21±0.10 (range, 0.10 to 0.48) logMAR in the Sensar group, 0.23±0.14 (range, 0 to 0.40) logMAR in the Akreos AO group, 0.18±0.10 (range, 0.0 to 0.30) logMAR in the Tecnis group, and 0.16±0.12 (range, 0.0 to 0.40) logMAR in the AcrySof IQ group (P=0.18). Corresponding figures for mean postoperative BSCVA were 0.11±0.10 (range, 0.0 to 0.25) logMAR, 0.12±0.9 (range, 0.0 to 0.30) logMAR, 0.08±0.08 (range, 0.0 to 0.18) logMAR, and 0.08±0.07 (range, 0.0 to 0.18) logMAR, in the study groups respectively (P=0.08). BSCVA ≥ 20/30 was observed in 82.4% of eyes in the Sensar group, 94.1% of the Akreos AO group, and 100% of cases in the Tecnis and AcrySof IQ groups (P=0.10). Data on refractive outcomes are presented in table 1.

Spherical aberration measured over a 4 mm pupil was significantly higher with Sensar and Akreos AO than the other two IOLs. Sensar and Akreos AO groups were comparable in this regard. The difference between the Tecnis and AcrySof IQ groups was significantly in favor of the former. Over a 6 mm pupil, comparisons between Sensar and Akreos AO, as well as that between Akreos AO, AcrySof IQ and Tecnis yielded comparable results. However, Sensar yielded results significantly inferior to those of Acrysof IQ and Tecnis. Spherical aberration significantly increased from 4 mm to 6 mm pupils in all four groups (Table 2) and there was no significant difference among the groups in this regard (P=0.18).

**Figure 1:**
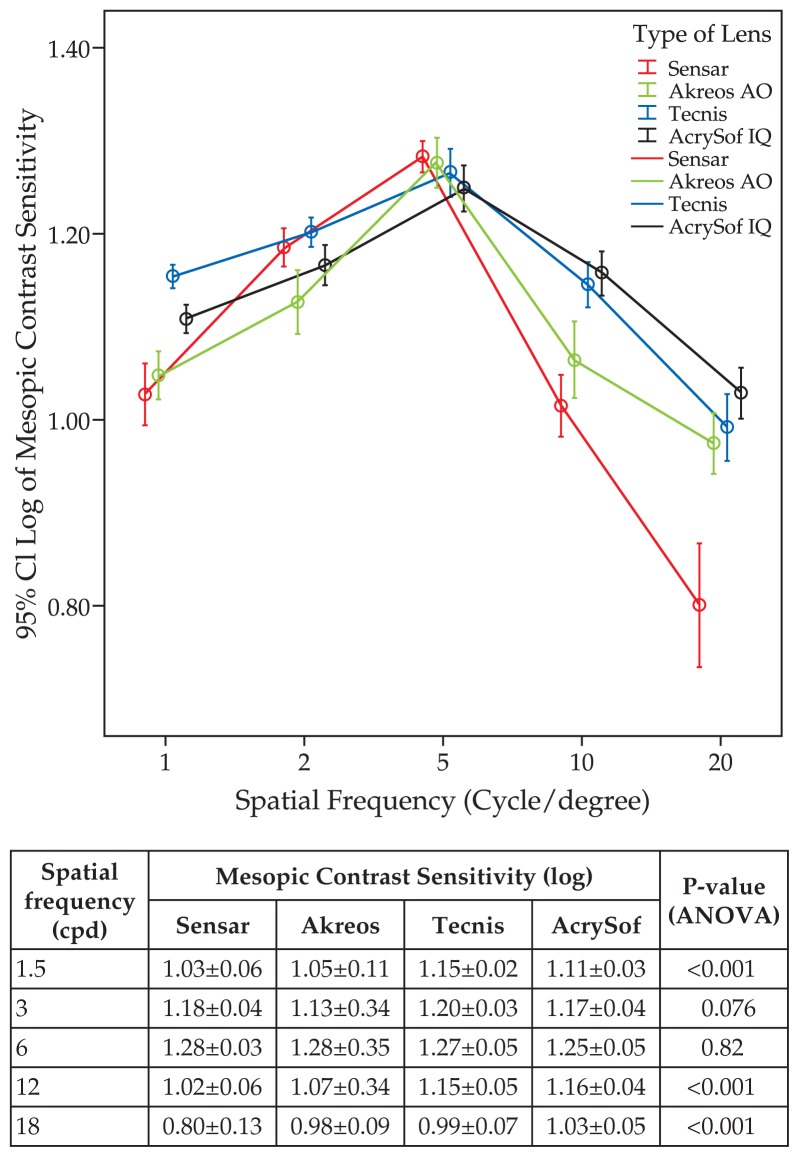
Postoperative contrast sensitivity function (log) at different spatial frequencies (cpd) measured under mesopic conditions.

**Figure 2:**
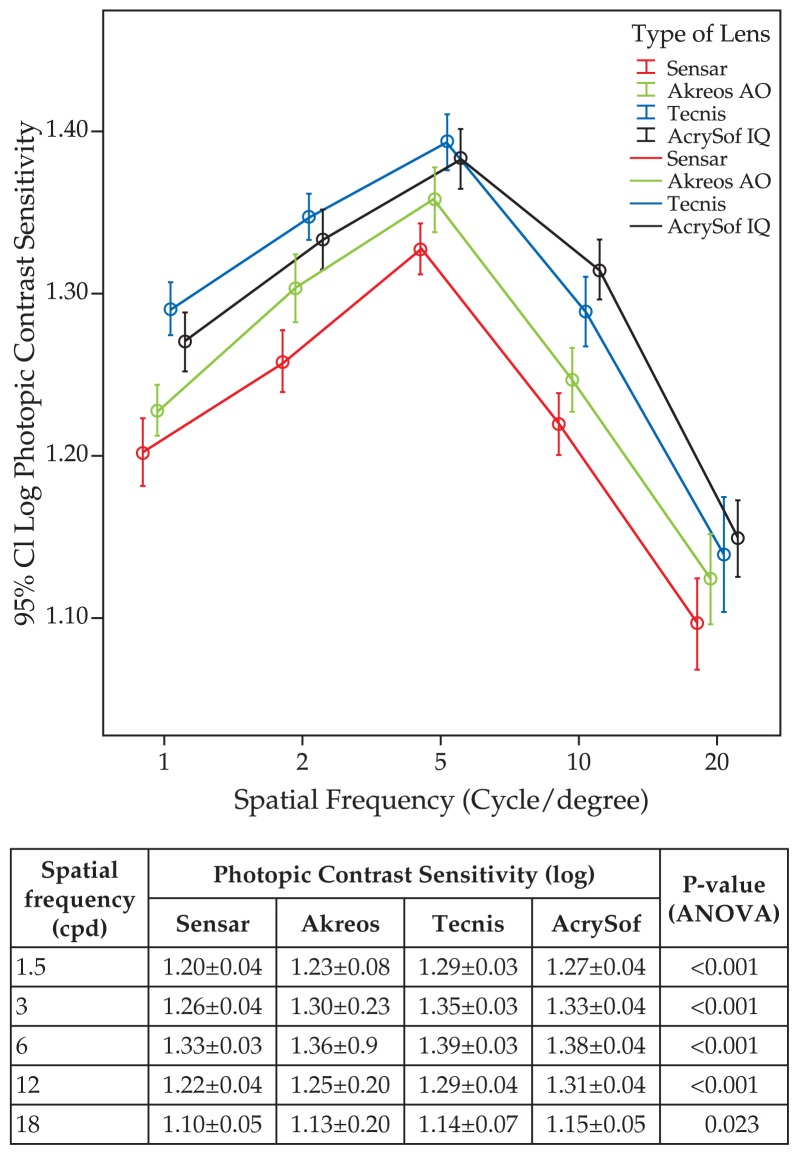
Postoperative contrast sensitivity function (log) at different spatial frequencies (cpd) measured under photopic conditions.

Figure 1 demonstrates that under mesopic conditions, CSF with Tecnis was significantly higher than that of the other two aspheric IOLs at spatial frequencies of 1.5 and 3 cpd. At 12 and 18 cpd, AcrySof IQ worked significantly better than the other three IOLs, while Tecnis performed better than Sensar and Akreos AO. Under photopic lighting conditions, the Tecnis group had significantly better results than the other three groups at 1.5, 3, and 6 cpd. At the same spatial frequencies, AcrySof IQ performed better than Sensar and Akreos AO. At spatial frequencies of 12 and 18 cpd, the Acrysof IQ worked better than the other three groups while Tecnis was superior to Sensar and Akreos AO (Fig. 2).

There were no intra- or postoperative complications in any of the cases and slitlamp examination revealed no IOL decentration or tilt.

## DISCUSSION

Conventional monofocal IOLs can introduce positive spherical aberration, adding approximately 0.08 μm (over a 4 mm pupil) to pre-existing aberrations caused by the cornea.^1^,^6^ Aspheric IOLs generate negative spherical aberration, leading to a smaller amount of postoperative spherical aberration as compared to spherical IOLs.^1^,^2^,^4^,^5^ The Akreos AO has aspheric surfaces to prevent the introduction of any spherical aberration. The AcrySof IQ SN60WF is given a modified posterior prolate design to induce negative spherical aberration equivalent to −0.20 μm over a 6 mm pupil. In the Tecnis ZA9003 IOL, this goal is achieved by creation of a modified prolate anterior surface to generate spherical aberration of −0.27 μm over a 6 mm pupil.

Advantages of aspheric IOLs over their spherical counterparts were demonstrated in the present study. The four study groups were comparable in terms of postoperative UCVA, BSCVA, and refractive error. However, visual function, including spherical aberration, and mesopic and photopic contrast sensitivity function, were significantly better with aspheric IOLs as compared to a spherical one. Among the four different IOLs implanted in this study, only one (AcrySof IQ) has blue light filtering properties. Some studies have shown that current UV-absorbing IOLs do not affect contrast sensitivity.^7^,^8^ Given that, differences in CSF observed in this study may be attributed only to higher order aberrations rather than the filtering properties of the yellow-tinted IOL.

Packer et al^9^,^10^ demonstrated that aspheric IOLs (Tecnis) provided significantly better contrast sensitivity at some spatial frequencies (3 and 6 cpd under photopic conditions and 1.5, 3, and 6 cpd under mesopic conditions). Mester et al^1^ observed that mesopic contrast sensitivity at low spatial frequencies was significantly better with Tecnis than with spherical IOLs. However, the aspheric IOL lost its advantages under photopic conditions. Similar results were reported by Rocha et al^11^, who observed significantly better contrast sensitivity with an aspheric IOL (AcrySof IQ) at 3 cpd spatial frequency under mesopic, but not photopic conditions. Comparing spherical IOLs (AcrySof SN60AT and Sensar AR40e) with aspheric IOLs (Tecnis Z9000 and AcrySof IQ SN60WF), Caporossi et al^12^ reported the maximum benefit of aspheric IOLs to occur under mesopic lighting conditions. In the mentioned study, there was no significant difference between the Tecnis Z9000 and AcrySof IQ SN60WF in terms of CSF under both mesopic and scotopic conditions.

We found that aspheric IOLs lost the advantage of enhancing visual performance to some extent under mesopic conditions as there was no significant difference between spherical and aspheric groups in terms of mesopic CSF measured at 6 cpd frequencies. This observation is parallel to the significant increase in spherical aberration from 4 mm to 6 mm pupils observed in all study groups. These findings are supported by two previous studies reporting no difference between aspheric and spherical IOLs in terms of mesopic contrast sensitivity with and without glare.^13^,^14^ This means that the benefit of aspheric IOLs depends on pupil size and their function deteriorates as the pupil dilates, resembling spherical IOLs. One possible explanation for this contradictory finding can be larger pupil diameters under mesopic conditions in the latter studies as well as ours. Another explanation can be the different measurement protocols used in different studies.

As the results of Tecnis and AcrySof IOLs were superior to Sensar and Akreos IOLs, it seems that the former IOLs can provide better postoperative visual function. The choice between Tecnis and AcrySof IQ, however, depends on corneal spherical aberrations. Considering the preoperative corneal spherical aberration, Packer et al^16^ concluded that the AcrySof IQ is suitable for corneal spherical aberration from +0.1 to +0.235 μm and the Tecnis Z9000 or Tecnis Z9002 is a better alternative with corneal spherical aberrations exceeding +0.235 μm. It is advisable to implant Tecnis for patients who tend to have a large pupil diameter under mesopic conditions.

In summary, Tecnis and AcrySof IQ can significantly reduce postoperative spherical aberrations and improve visual function as compared to Sensar and Akreos AO. However, their performance depends on pupil diameter and under dim conditions, their function deteriorates to some extent. Providing relatively better contrast sensitivity function under mesopic conditions, Tecnis appears to be an appropriate choice for patients with a large mesopic pupil diameter.

## Figures and Tables

**Table 1:** Intraocular lens power, spherical equivalent refractive error and keratometric astigmatism in the study groups

	Sensar	Akreos AO	Tecnis	AcrySof IQ	P-value*
Mean IOL power (D)	22.8 ± 3.5	23.1 ± 4.1	21.9 ± 1.7	22.5 ± 2.6	0.45
Mean spherical equivalent refractive error (D)	−0.27 ± 0.20	−0.19 ± 0.16	−0.24 ± 0.18	−0.18 ± 0.14	0.51
Mean keratometry (D)	43.76 ± 2.1	42.95 ± 1.8	44.23 ± 2.4	44.82 ± 3.1	0.24

D, diopter;

* ANOVA

**Table 2:** Spherical aberration measured over 4 mm and 6 mm pupils in the study groups

Type of IOL	Spherical aberration (Z_4_^0^; μm)		P-value*
	4 mm pupil	6 mm pupil		
Sensar	0.35 ± 0.14	0.44 ± 0.15	<0.001
Akreos AO	0.29 ± 0.09	0.33 ± 0.08	0.02
Tecnis	0.14 ± 0.10	0.30 ± 0.18	0.013
AcrySof IQ	0.18 ± 0.11	0.27 ± 0.10	<0.001

P-value**	<0.001	0.005	

* paired t-test

** ANOVA
